# First Clarification of the Mechanism of Action of the Apple Glycosyltransferase MdUGT91AJ2 Involved in the Detoxification Metabolism of the Triketone Herbicide Sulcotrione

**DOI:** 10.3390/plants13131796

**Published:** 2024-06-28

**Authors:** Aijuan Zhao, Xiao Teng, Yingxin Ma, Lijun Mu, Shibo Han, Shumin Wang, Kang Lei, Lusha Ji, Pan Li

**Affiliations:** 1 State Key Laboratory for Macromolecule Drugs and Large-Scale Manufacturing, School of Pharmaceutical Sciences, Liaocheng University, Liaocheng 252059, China; zaj15105403245@163.com (A.Z.); m13021553065@163.com (Y.M.); mlj15215354741@163.com (L.M.); hshibo1999@163.com (S.H.); 13686366329@163.com (S.W.); leikang@lcu.edu.cn (K.L.); 2 Rizhao Academy of Agricultural Science, Rizhao 276500, China; teng-xiao-rzaas@hotmail.com

**Keywords:** glycosyltransferase, MdUGT91AJ2, triketone herbicides, sulcotrione, detoxification metabolism

## Abstract

Sulcotrione is a member of triketone herbicides, a class of HPPD (4-hydroxyphenylpyruvate dioxygenase) inhibitors with broad-spectrum herbicidal activity. Modifications of glycosylation mediated by glycosyltransferases (GT) are involved in plant detoxification. In this study, we analyzed chip data published online and found that eight glycosyltransferases from group A of the apple glycosyltransferase family 1 may be involved in the metabolic mechanism of detoxification of triketone herbicides. To verify this prediction, we induced apple seedlings with six types of triketone herbicides, and then detected the expression levels of eight glycosyltransferase genes through real-time PCR. We found that triketone herbicides induced up-regulation of eight glycosyltransferase genes to varying degrees, with *MdUGT91AJ2* being the most significantly up-regulated by sulcotrione-induced glycosyltransferase gene expression. Then, through in vitro enzymatic reactions and HPLC identification of glycoside substrates, it was found that the glycosyltransferase MdUGT91AJ2 had the highest specific enzyme activity against the triketone herbicide sulcotrione. Furthermore, the in vivo mechanism of the glycosyltransferase MdUGT91AJ2 in the detoxification metabolism of sulcotrione was further validated by overexpressing the strain in the plant. HPLC analysis showed that the content of sulcotrione glycosides in the overexpressing strain of *MdUGT91AJ2* was significantly higher than that in the wild type. This result indicated that the apple glycosyltransferase MdUGT91AJ2 can still glycosylate and modify sulfotrione in plants, and participate in its detoxification metabolism. In summary, this study identified for the first time a novel apple glycosyltransferase MdUGT91AJ2 and elucidated its mechanism of action in the detoxification and metabolism of the triketone herbicide sulfotriene.

## 1. Introduction

**Figure 1:**
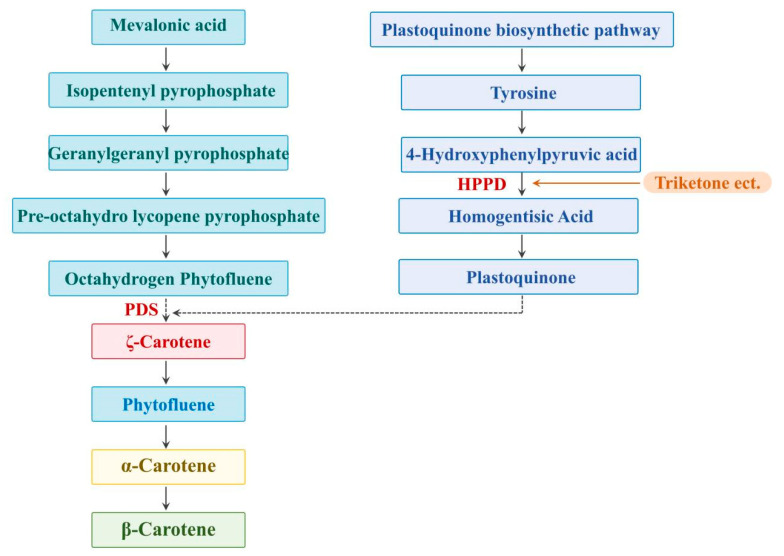
Mechanism of herbicidal action of triketones.

Triketone herbicides are another class of HPPD (p-hydroxyphenylpyruvate dioxygenase) inhibitors developed by Jellicam after pyrazole herbicides. Their action is characterized by broad-spectrum herbicidal activity and can be used both pre-emergence and postemergence. Currently, several varieties have been successfully developed, including sulcotrione, furazosulfuron, bicyclosulfuron, nitrosulfuron, cycloheximide, and flupyradifuron [1]. The target of the action of the triketone herbicides is the HPPD enzyme, which exists in a wide range of organisms, and is the key enzyme in the synthesis of plastoquinone and α-tocopherol in the plant (see Figure 1). HPPD inhibitors inhibit the synthesis of HPPA (4-hydroxyphenylpyruvic acid) into HGA (homogentisate), which leads to the blockage of the synthesis of plastoquinone, and then impedes the catalysis of the phytoene desaturase (PDS), affecting the biosynthesis of carotenoids. The decrease in carotenoid synthesis leads to a lack of protective pigments in chlorophyll, resulting in weeds yellowing or death due to bleaching symptoms [2,3]. Sulcotrione is a foliar herbicide that can also be absorbed through the root system causing the weeds to discolor quickly and die slowly after application. However, the mode of action is still not fully understood and it is likely that chlorophyll synthesis is directly affected, acting finally on carotenoid synthesis [4]. Pyrazosulfuron-methyl inhibits the synthesis of pigments essential for plant growth, which is absorbed and rapidly transmitted through roots, stems, young shoots, and leaves, ultimately affecting the biosynthesis of carotenoids, and the weed is subjected to whitening of the foliage followed by necrosis of the meristematic tissue [5]. Dicyclosulfocarbazone, which has a bicyclic and phenylthioether structure, is absorbed by weed roots and stems, and then transported to the whole plant, where it affects the synthesis of plastoquinone through inhibition of the HPPD, and then the action of plastoquinone on octahydroxy phytoene desaturase (PDS), which ultimately affects the biosynthesis of carotenoids and bleaching of the foliage [6]. Cyclosulfamuron is effective at controlling major broadleaf grasses and some grassy weeds and is readily conducted in the xylem and phloem of plants; it is both touched and persistent [7]. Cyclosulfamuron is mainly used in maize (*Zea mays* L.) fields and blocks isoprenoid quinone biosynthesis in the plant, causing green loss, discoloration, tissue necrosis, and eventual death within two weeks [8]. Flumioxazin can cause symptoms of bleaching and eventual death of plant meristematic tissues by inhibiting carotenoid biosynthesis [9].

Herbicides of the triketone group are widely used in agricultural fields such as maize (*Zea mays* L.) to control grass weeds and broadleaf weeds [10]. The herbicide is weakly acidic, its aqueous solution is highly stable, not easy to photolyze and volatilize, and it is widely used in large quantities, which can be harmful to the ecological environment of the soil when it is used inappropriately or repeated in large quantities over a long period of time [11]. Despite the low toxicity of sulcotrione itself, it has been shown that the metabolites of sulcotrione in the environment are higher than the parent itself, and studies on onion root meristematic tissues under light conditions found that the cytotoxicity and genotoxicity of sulcotrione photolysis products were significantly higher than those of the sulcotrione parent [12]. In addition to the product CMBA (2-chloro-4-meta phenylbenzoic acid), sulfoxaflor photolysis also generates a class of cyclisation product CP (cyclization product) [13]. Halle et al., compared the toxicity of sulcotrione, CMBA, and CP to microbial bacteria (*Vibrio fischeri*), algae (*Pseudokirchneriella subcapitata*), and protists (*Tetrahymena pyriformis*), respectively. The results indicated that sulcotrione produces higher toxicity to algae and greater CP toxicity to bacteria (*Vibrio fischeri*) and protists (*Tetrahymena pyriformis*) [14]. The toxicity of the photolytic mixture of sulcotrione is higher than that of the parent and CMBA, therefore, the role of its cyclized metabolites in the environmental behavior and risk assessment of sulcotrione deserves continued attention [15]. Pesticides enter the soil system and their degradation involves a complex series of chemical processes that can be biotransformed or abiotransformed [16]. Bioremediation, also known as biomanagement, refers to the use of the life metabolic activities of organisms to absorb, degrade, and transform pollutants in the environment so that the polluted environment can be improved [17]. It can be divided into microbial remediation, phytoremediation, and animal remediation according to the type of organisms [18]. Phytoremediation mainly involves remediation of pollutant residues in the environment through plant uptake, plant extraction, plant volatilization, and root secretions in association with inter-root microorganisms [19]. Qu et al. have shown that in order to cope with the harmful effects of exogenous toxin herbicides, the plant body has developed a series of detoxification mechanisms to convert toxic exogenous toxins into nontoxic substances during the long-term evolutionary process, which in turn reduces the damage to plant cells [20]. The detoxification mechanism consists of two phases: the first phase is mainly an activation reaction, i.e., hydrolysis or oxidation [21]; and the second phase is mainly a conjugation reaction, which usually involves linking the metabolite or heterologue activated in the first phase directly to an endogenous hydrophilic molecule, e.g., glucose, malonate, or glutathione, by means of covalent bonding [22].

Plant UDP-glycosyltransferases are an important class of cytochrome P450 enzyme coenzymes involved in a variety of biological processes such as plant growth and development, secondary metabolism, and environmental adaptation. Numerous studies have shown that glycosyltransferases can participate in the detoxification reactions of organisms by transferring enzyme glucose or other sugar groups [23,24,25]. UGTs play an important role by facilitating the binding of harmful substances and limiting the effects of harmful substances on normal physiological and metabolic processes [23]. Blomstedt et al. succeeded in identifying a sorghum (*Sorghum bicolor* (L.) *Moench*) glycosyltransferase gene, *UGT85B1*, and found that the mutation in this gene resulted in reduced plant vigor, dwarf plants, poor roots development, low fertility, and no production of the toxic substance dhurrin. Further investigation of the mechanism revealed that the reason why this mutant did not show toxicity was sorghum did not have a feedback mechanism to inhibit the initial steps of dhurrin biosynthesis when lacking the glucosyltransferase activity required to complete dhurrin synthesis [24]. Su et al. proposed a TCP (trichlorophenol) phytoremediation system based on sugar conjugation by overexpressing poplar (*Populus euphratica Oliv*) UDP-glucosyltransferase. It was found that among the many glycosyltransferases, PtUGT72B1 had the highest binding activity to TCP, and its overexpression of *Arabidopsis thaliana* lines showed significantly enhanced tolerance to 2,4,5-TCP and 2,4,6-TCP [25]. Although the involvement of plant glycosyltransferases in the detoxification process has been reported in some cases, the involvement of apple (*Malus* × *domestica*) glycosyltransferases in the metabolic mechanism of detoxification of triketone herbicides has hardly been studied. Therefore, in this study, a new apple (*Malus* × *domestica*) glycosyltransferase, MdUGT91AJ2, was identified for the first time, and its mechanism of action in the detoxification metabolism of the triketone herbicide sulcotrione was clarified, which will provide scientific data for the reduction of the potential risks of pesticides to ecosystems, crops, animals, and humans.

## 2. Results

### 2.1. Induction of mRNA Expression Level of Apple Glycosyltransferase Gene by Triketone Herbicides

**Figure 2:**
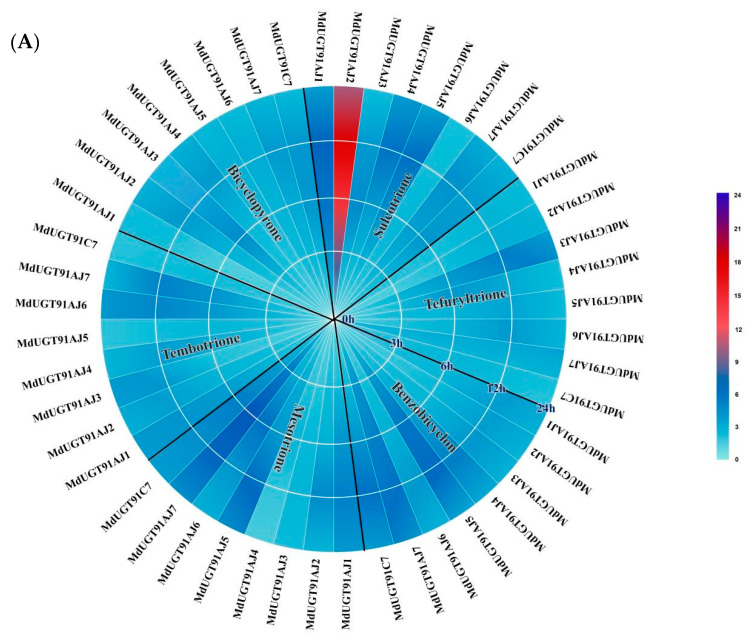

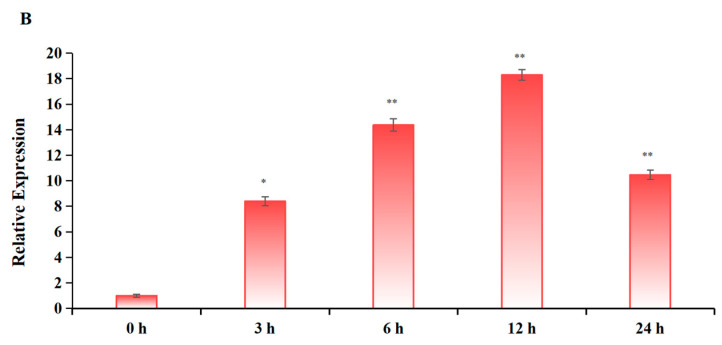
Real-time PCR detection of six triketone herbicides, as sulcotrione, tefuryltrione, benzobicyclon, mesotrione, tembotrione, and bicyclopyrone after induction for 0, 3, 6, 12 and 24 h. In apple (*Malus* × *domestica*) glycosyltransferase family 1, 8 glycosyltransferase genes in group A of *MdUGT91AJ1* (*MD02G1083600*), *MdUGT91AJ2* (*MD02G1084000*), *MdUGT91AJ3* (*MD15G1211400*), *MdUGT91AJ4* (*MD10G1101200*), *MdUGT91AJ5* (*MD02G1083100*), *MdUGT91AJ6* (*MD02G1083300*), *MdUGT91AJ7* (*MD02G1083800*), *MdUGT91C7* (*MD08G1200700*) mRNA expression levels. (**A**) Real-time PCR to detect the mRNA expression levels of eight glycosyltransferase genes in group A of apple (*Malus* × *domestica*) glycosyltransferase family 1 induced by each of the six triketone herbicides; (**B**) real-time PCR to detect the mRNA expression levels of the apple (*Malus* × *domestica*) glycosyltransferase gene, *MdUGT91AJ2*, induced by the triketone herbicide sulcotrione. Note: * *p* < 0.05, ** *p* < 0.01, *n* = 3.

We analyzed the microarray data published online (https://www.ncbi.nlm.nih.gov/, accessed on 10 December 2023) and found that eight glycosyltransferases in group A of apple (*Malus* × *domestica*) glycosyltransferase family 1, *MdUGT91AJ1* (*MD02G1083600*), *MdUGT91AJ2* (*MD02G1084000*), *MdUGT91AJ3* (*MD15G1211400*), *MdUGT91AJ4* (*MD10G1101200*), *MdUGT91AJ5* (*MD02G1083100*), *MdUGT91AJ6* (*MD02G1083300*), *MdUGT91AJ7* (*MD02G1083800*), and *MdUGT91C7* (*MD08G1200700*) may be involved in the mechanism of triketone herbicide detoxification metabolism. To verify the prediction, we exogenously sprayed apple (*Malus* × *domestica*) seedlings with six triketone herbicides, sulcotrione, tefuryltrione, benzobicyclon, mesotrione, tembotrione, and bicyclopyrone, extracted the RNAs, and reverse-transcribed them into cDNAs, and then detected the expression levels of eight glycosyltransferase genes via real-time PCR; the results are shown in Figure 2. Triketone herbicides induced the up-regulation of the expression of eight glycosyltransferase genes to varying degrees (Figure 2A, Table S4), with sulcotrione inducing the most significant up-regulation of the glycosyltransferase gene, *MdUGT91AJ2* (* *p* < 0.05, ** *p* < 0.01) (Figure 2B).

### 2.2. In Vitro Enzyme Activity Assay of Apple Glycosyltransferase Glycosylated Modified Triketone Herbicides

**Figure 3:**
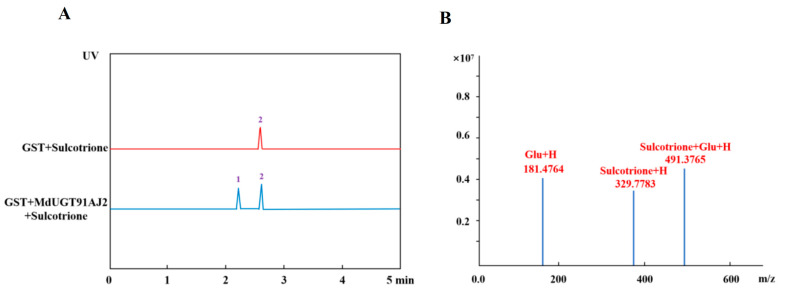
HPLC detection of MdUGT91AJ2 glycosylation modification substrate sulcotrione. (**A**) HPLC detection of MdUGT91AJ2 glycosylation modification product sulcotrione glucoside; (**B**) mass spectrometry detection of MdUGT91AJ2 glycosylation modification product sulcotrione glucoside molecular weight. Note: 1: sulcotrione; 2: sulcotrione glucoside.

In the above study, we found that triketone herbicides induced different degrees of mRNA levels of eight glycosyltransferase genes in group A of apple (*Malus* × *domestica*) glycosyltransferase family 1, which are enzymes in plants that can transfer sugar groups to different small molecule compounds. For this reason, we obtained eight glycosyltransferase proteins successively through prokaryotic expression vector construction and in vitro enzyme-protein purification system, performed in vitro enzymatic reactions against six triketone herbicides, and identified glycosidic substrates by HPLC, and found that glycosyltransferases were selective and preferential to triketone herbicides (Table 1). The glycosyltransferase MdUGT91AJ2 had the highest specific enzyme activity for the triketone herbicide sulcotrione compared to other glycosylation modifications, i.e., MdUGT91AJ2 was the most active for sulcotrione glycosylation modification (Figure 3A,B).

### 2.3. Analysis of the Expression Pattern of MdUGT91AJ2 in Different Tissue Sites

**Figure 4:**
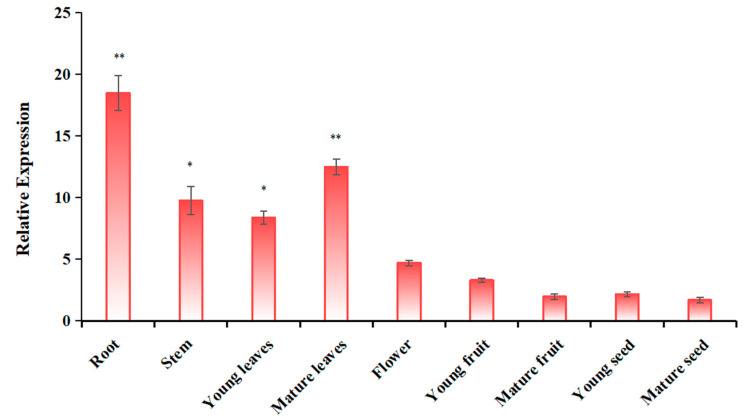
Real-time PCR to detect the expression of different parts of apple glycosyltransferase gene MdUG91AJ2. Note: compared with the mature seed group, * *p* < 0.05, ** *p* < 0.01, *n* = 3.

In order to further understand the tissue expression pattern of *MdUGT91AJ2*, we detected the gene expression level of *MdUGT91AJ2* in different parts of apple (*Malus* × *domestica*) via real-time PCR and the results are shown in Figure 4. The expression level of *MdUGT91AJ2* varied considerably in different parts of the apple (*Malus* × *domestica*), with the highest expression level in the root system, followed by that in mature leaves, while in mature seeds the expression level was the lowest. Combined with the above results that MdUGT91AJ2 was able to glycosylate and modify sulcotrione herbicide, we speculated that MdUGT91AJ2 may play a detoxification metabolism role in the root system and mature leaves.

### 2.4. Glycosylation of Apple Glycosyltransferase MdUG91AJ2 In Vivo in Plant to Modify Sulforaphane Enzyme Activity Assays

**Figure 5:**
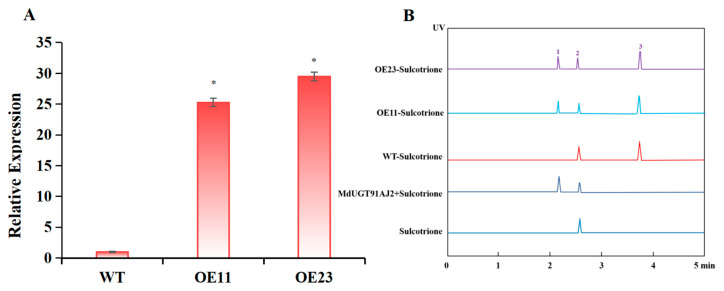
*MdUGT91AJ2* overexpression lines obtained and sulcotrione glycoside content assayed. (**A**) Detection of *MdUGT91AJ2* gene expression level in overexpression lines *OE11* and *OE23*; (**B**) determination of sulcotrione and sulcotrione glycoside content in *MdUGT91AJ2* overexpression lines *OE11* and *OE23*, 1: sulcotrione glycoside, 2: sulcotrione, 3: internal reference. Note: * *p* < 0.05, *n* = 3.

In the above study, we demonstrated that MdUGT91AJ2 can glycosylate sulcotrione in vitro and its activity was much higher than that of the other seven glycosyltransferases; MdUGT91AJ2 was also able to glycosylate sulcotrione in plants. To this end, we overexpressed *MdUGT91AJ2* into apples (*Malus* × *domestica*) and obtained transgenic overexpression lines *MdUGT91AJ2-OE11* and *MdUGT91AJ2-OE23* after screening (Figure 5A). After exogenous spraying of sulcotrione, HPLC determination of sulcotrione glycoside content in each strain. The result revealed a significant increase in sulcotrione glycoside content in the overexpression lines (Figure 5B). This study suggested that the apple (*Malus* × *domestica*) glycosyltransferase MdUG91AJ2 can still glycosylate modified sulcotrione in plants to participate in detoxification metabolism.

## 3. Discussion

With the gradual development of agriculture, herbicides have become one of the most common weed control tools in the world. Triketones, derived chemically from a natural phytotoxin (leptospermone), are a good example of allelochemicals as lead molecules for the development of new herbicides. Triketone herbicides are the latest generation of herbicides developed to inhibit a novel key enzyme involved in carotenoid biosynthesis, HPPD (4-hydroxyphenylpyruvate dioxygenase herbicide) [26]. HPPD belongs to α-ketate dependent, nonsemi-containing, and Fe^2+^ dependent enzymes that are important enzymes in tyrosine degradation. They can catalyze the formation of the aromatic precursor HGA (homogentisic acid) of plasmic quinones and vitamin E. The reaction involves decarboxylation, substituent migration, and aromatic oxidation in a single catalytic cycle [27]. Its inhibition leads to the consumption of plant plastid quinones and vitamin E, leading to bleaching syndrome, which is crucial for the discovery of new bleaching herbicides [28]. The decrease in carotenoid levels in weeds leads to rapid degradation of chlorophyll and visible bleaching of sensitive plants, which is due to uncontrolled free radical production and chlorophyll oxidation altering cell structure [29]. Corn growers often use β-triketone herbicides that are used to prevent the growth of weeds in the field. This has brought a lot of convenience to farmers. However, the application of herbicides can pollute the atmosphere and be intercepted and absorbed by weeds and crops. Plants containing pesticide residues may reach the soil during the crop cycle or after harvest, causing pollution of soil, surface water, and groundwater, posing a further threat to the ecological environment [30].

The pollution of toxic substances in the environment has become a concern for agricultural countries. Photolysis is an important way for pesticides to dissipate on crops, so people achieve ideal results by increasing the use of pesticides [31]. Sulcotrione is a trione herbicide used to control dicotyledonous weeds in maize cultivation. These herbicides are very potent for the selective pre- and in some cases postemergence control of a wide range of broadleaf and grass weeds in maize (*Zea mays* L.) and rice (*Oryza sativa*). It can rapidly photolyze on plant leaves and produce two main light products: oxoanthane-1,9-dione-3,4-dihydro-6-methylsulfonyl (XDD) and 2-chloro-4-meta phenylbenzoic acid (CMBA) [32]. However, an increase in the concentration of XDD and CMBA can significantly inhibit the mitosis of plant cells, leading to a continuous increase in cells with chromosomal abnormalities [13]. At the same time, it can also damage the normal growth of crops, so it is necessary to study an antidote to herbicides.

Previous studies have found that glycosylation modifications led by glycosyltransferases in plants are involved in detoxification [33,34,35]. Huang et al. found that the glycosyltransferase UGT91C1 can significantly reduce the toxicity of the triketone herbicide sulcotrione, enhance the resistance of arabidopsis to sulcotrione, and thus reduce its own damage and chlorophyll destruction [33]. Li et al. found that the glycosyltransferase Group I family has a significant detoxification effect on (acetyl CoA carboxylase) inhibitor herbicides, with the glycosyltransferase MdUGT83K2 showing the most significant detoxification effect on the ACCase herbicide QPP-7 [34]. Zhang et al. found that the glycosyltransferase Group D family has a significant detoxification effect on (ALS) inhibitor herbicides. Among them, the glycosyltransferase MdUGT73CG22 has the strongest specificity and most significant detoxification effect on the ALS herbicide nicosulfuron [35]. In this study, we demonstrated that the glycosyltransferase MdUGT91AJ2 in apples (*Malus* × *domestica*) can glycosylate the herbicide sulcotrione in vitro and in vivo, and this enzyme had substrate specificity for carotene. After using the overexpressed strains, we also found that the glycosyltransferase MdUGT91AJ2 enhanced plant resistance to such herbicides. In summary, this work not only revealed the role of a new specific glycosyltransferase in apples (*Malus* × *domestica*) in detoxifying the triketone herbicide sulcotrione, but also opened a new door for addressing the use of herbicides in apple (*Malus* × *domestica*) cultivation and development.

## 4. Materials and Methods

### 4.1. Plant Material

Roots, stems, leaves, flowers, fruits, and seeds of a 10-year-old “*Gala*” apple (*Malus* × *domestica*) tree (*Malus* × *domestica Borkh.*) planted in the School of Life Sciences of Shandong Agricultural University were selected to investigate the expression characteristics of MdUGT91AJ2 in different tissues of apple (*Malus* × *domestica*). After sampling, they were quickly frozen in liquid nitrogen and stored for use at −80 °C. The apple (*Malus* × *domestica*) seedling material used in this study was “*Gala*” apple (*Malus* × *domestica*) seedlings, which were bred and preserved in our laboratory. The apple (*Malus* × *domestica*) seedlings were cultured in MS (Murashige and Skoog) medium and the overexpression of MdUGT91AJ2 transgenic seedlings were cultured in MS medium containing 25 mg/L Kanamycin. After 2 weeks, the seedlings were transplanted to a mixed (substrate: vermiculite: perlite 4:1:1) nutrient substrate in an incubator at 24 °C with 16 h of light/8 h of darkness. The planting conditions of plant materials referred to reference [36], but there were some changes. After three weeks, the seedlings were used for experiments.

### 4.2. Exogenous Treatment of Apple Seedlings with Triketone Herbicides

Apple (*Malus* × *domestica*) seedlings, which had been normally cultured for 5 weeks, were randomly divided into six groups and sprayed with 10 µmol/L sulcotrione, tefuryltrione, benzobicyclon, mesotrione, tembotrione, and bicyclopyrone (Table 2) for different times of treatment (0, 3, 6, 12, 24 h) and immediately liquid nitrogen snap-frozen and stored at −80 °C for subsequent RNA extraction.

### 4.3. Real-Time PCR

RNA extraction: plant material was subjected to RNA extraction using an RNA extraction kit (DP437, Tiangen Biochemical Science and Technology Co. Ltd, Beijing, China).

cDNA reverse transcription: cDNA first strand synthesis was performed using PrimeScript^TM^ RT reagent Kit with a gDNA Eraser kit (DRR047A, Bao Bioengineering Co., Ltd, Dalian, China).

Real-time PCR: The UltraSYBR Mixture kit (CW0659S, Kangwei Century Technology Co., Ltd., Beijing, China) was used, and the fluorescence quantitative PCR instrument model was the BIO-RAD IQ5. The primer sequences are shown in Table S1. A total of 20 μL of the PCR reaction system was composed of: 2× Ultra SYBR Mixture 10.0 μL, Forward primer (10 μmol·L^−1^) 1.0 μL, Reverse primer (10 μmol·L^−1^) 1.0 μL, cDNA 1.0 μL, and ddH_2_O 7.0 μL. The reaction procedure: predenaturation at 94 °C for 10 min; denaturation at 94 °C for 15 s, annealing at 56 ℃ for 15 s, 40 cycles, and then the data were analyzed via 2^−∆∆^CT algorithm.

### 4.4. Expression Vector Construction

Download the coding sequences of the eight glycosyltransferase genes( *MdUGT91AJ1 (MD02G1083600)*; *MdUGT91AJ2 (MD02G1084000)*; *MdUGT91AJ3 (MD15G1211400)*; *MdUGT91AJ4 (MD10G1101200)*; *MdUGT91AJ5 (MD02G1083100)*; *MdUGT91AJ6 (MD02G1083300)*; *MdUGT91AJ7 (MD02G1083800)*, *MdUGT91C7 (MD08G1200700))* from the apple genome database GDDH13 v1.1 (Table S2). Full-length primers for MdUGT91AJ2 gene were designed (Table S1). The coding region fragments of MdUGT91AJ2 gene were cloned using ‘Gala’ cDNA as a template. PCR amplification was performed using the high-fidelity 2×PCR Solution PrimeSTAR^TM^ HS Premix (DR040A, Bao Bioengineering Co., Ltd., Dalian, China). The PCR products were electrophoresed, recovered, and ligated into T vector (pEASYBlunt Zero, Beijing Total Gold), and the positive clones were selected after PCR verification of the bacterial liquid and sent to Shanghai Sangong Biotechnology Co. Ltd. for sequencing. One hundred percent of the sequencing was correctly performed and the products of the digestion were ligated into the same endonuclease-digested pGEX-2T [37] and PBI121 vectors [38], respectively. After verification via bacteriological PCR and double enzymatic digestion, the positive recombinant plasmids MdUGT91AJ1-pGEX-2T, MdUGT91AJ2-pGEX-2T, MdUGT91AJ3-pGEX-2T, MdUGT91AJ4-pGEX-2T, MdUGT91AJ5-pGEX-2T, MdUGT91AJ6-pGEX-2T, MdUGT91AJ7-pGEX-2T, MdUGT91C7-pGEX-2T, and MdUGT91AJ2-pBI121 were transformed into E. coli BL-21 and R. rhizogenes strain K599 for subsequent protein expression and transgenic strain screening.

### 4.5. Glycosyltransferase Protein Purification and In Vitro Enzyme Activation Reaction

The *E. coli BL21* strain containing positive recombinant plasmid was inoculated into 2 mL LB medium containing Amp^+^ antibiotic, and incubated at 37 °C with shaking overnight. It was then diluted into 100 mL triangular flasks according to the ratio of 1:20, and shaking cultivation continued for about 2 h. When the OD_600_ value was about 0.6, the expression was induced by adding the final concentration of 1 mmol·L^−1^ IPTG, and the induction temperature was 20 °C; the induction incubation time was 16 h. Centrifugation was performed at 4200 rpm·min^−1^ for 10 min, 10 mL of the bacterial bodies was collected, and 1 mL of 1×PBS buffer was used for the incubation. A total of 1 mL of 1×PBS buffer was used to resuspend the organisms, and the organisms were treated via repeated freeze-thawing method, centrifuged at 12,000 rpm·min^−1^ for 15 min, and then all the supernatant was taken. The recombinant proteins were purified using a His-tag Protein Purification Kit Tagged Protein Purification Kit (BYX8228C, Beiyuanxin Biotechnology Co., Ltd., Changzhou, China), according to the instructions. The amino acid sequences of the eight glycosyltransferases in this study are shown in Table S3.

In vitro enzymatic reaction: 100 µL in vitro enzymatic reaction system containing 10 µL 0.5 M Tris-HCl (pH 8.0), 5 µL 50 mM MgSO_4_, 5 µL 200 mM KCl, 2.5 µL 0.1 M UDP-glucose, 1 µL 10% β-mercaptoethanol, 1 µL 100 mM sulcotrione, 2 µL MdUGT91AJ2 enzyme protein, and 73.5 µL ddH_2_O [34].

### 4.6. Determination of Triketone Herbicide Glycosides via HPLC

Glycoside extraction: 5-week-old apple (*Malus* × *domestica*) seedlings treated with 10 µM sulcotrione, tefuryltrione, benzobicyclon, mesotrione, tembotrione, and bicyclopyrone for 12 h were milled to powder with liquid nitrogen. They were transferred to 10 mL of 80% methanol in a triangular vial and 2 µL of endosulfan (Piclorom) was added. The samples were placed in a shaker for 6 h at 60 r·min^−1^ and shaken slowly. Then, they were transferred to a rotary evaporator for vacuum evaporation and the residual powder on the inner wall of the centrifuge tube was dissolved with 1 mL of methanol solution and centrifuged at 15,000 rpm for 20 min; the glycosides were detected via HPLC [39].

HPLC conditions: ACQUITY UPLC BEH Amide column (100 mm × 2.1 mm × 1.7 μm), column temperature 40 °C, temperature in the sample chamber 10 °C, injection volume 5.0 μL, mobile phase A was 0.1% ammonia-acetonitrile, and mobile phase B was 1 mmol/L ammonium acetate (containing 0.1% ammonia) at a flow rate of 0.3 mL·min^−1^. The gradient elution procedure was 0~1.0 min, 100% A; 1.0~2.5 min, 100% ~60% A; 2.0~4.0 min, 60% ~100% A; and 4.0~5.0 min, 100% A. The elution was carried out at a flow rate of 0.3 mL·min^−1^.

Mass spectrometry conditions: ESI source, positive ion mode, multiple reaction detection scanning (MRM), capillary voltage 3.5 kV, cone-well voltage 35 V, ion source temperature 100 °C, desolvent gas temperature 300 °C, cone-well backblast nitrogen flow rate of 50 L·h^−1^, desolvent gas flow rate of 600 L·h^−1^, photomultiplier voltage of 650 V, and collision chamber vacuum 0.076 Pa. The conditions were the same as those of HPLC. The elution method was the same, and the surface-induced decomposition intensity was 30 eV.

### 4.7. Transgenic Apple Seedling Screening

The apple genetic transformation experiment used “*Gala*” apple (*Malus* × *domestica*) seedlings as materials, and obtained the line “GL-3” group-cultured seedlings with strong regeneration ability and high transformation efficiency. The leaves of “GL-3” were transformed with Agrobacterium tumefaciens carrying the sequence of MdUGT91AJ2, and the transgenic apple (*Malus* × *domestica*) lines *OE11* and *OE23* were obtained. The specific operation was referred to in reference [40,41], which included the steps of preparation of bacterial solution, infiltration, bacterial washing, screening, and identification. After obtaining the positive strains, they were propagated, rooted, and transplanted to soil, and then grown to a certain stage for subsequent tests.

### 4.8. Data Statistics and Analyses

The experimental data are expressed as “mean ± standard error.” The data were processed and statistically analyzed using Excel 2019 and SPSS 21.0, and graphs were produced; the difference was tested for significance using the Least Significant Difference (LSD) method (* *p* < 0.05, **p < 0.01).

## Figures and Tables

**Table 1:** Specific enzyme activity determination of eight apple glycosyltransferases against triketones herbicides.

Name	Substrates	Specific Activity (nkat/mg Protein)
MdUGT91AJ1	Sulcotrione	1.24 ± 0.19
	Tefuryltrione	ND
	Benzobicyclon	ND
	Mesotrione	ND
	Tembotrione	ND
	Bicyclopyrone	ND
MdUGT91AJ2	Sulcotrione	3.56 ± 0.25
	Tefuryltrione	ND
	Benzobicyclon	ND
	Mesotrione	ND
	Tembotrione	ND
	Bicyclopyrone	ND
MdUGT91AJ3	Sulcotrione	ND
	Tefuryltrione	0.36 ± 0.11
	Benzobicyclon	ND
	Mesotrione	ND
	Tembotrione	ND
	Bicyclopyrone	ND
MdUGT91AJ4	Sulcotrione	0.76 ± 0.20
	Tefuryltrione	ND
	Benzobicyclon	ND
	Mesotrione	ND
	Tembotrione	ND
	Bicyclopyrone	ND
MdUGT91AJ5	Sulcotrione	ND
	Tefuryltrione	ND
	Benzobicyclon	ND
	Mesotrione	ND
	Tembotrione	ND
	Bicyclopyrone	ND
MdUGT91AJ6	Sulcotrione	ND
	Tefuryltrione	ND
	Benzobicyclon	ND
	Mesotrione	0.31 ± 0.04
	Tembotrione	ND
	Bicyclopyrone	ND
MdUGT91AJ7	Sulcotrione	ND
	Tefuryltrione	ND
	Benzobicyclon	ND
	Mesotrione	0.45 ± 0.08
	Tembotrione	ND
	Bicyclopyrone	ND
MdUGT91C7	Sulcotrione	ND
	Tefuryltrione	ND
	Benzobicyclon	ND
	Mesotrione	0.39 ± 0.07
	Tembotrione	ND
	Bicyclopyrone	ND

Note: ND, none detected.

**Table 2:** Specific information on triketone herbicides.

Chemical Compound	Structure Name	Structural Formula	Manufacturer	CAS
Sulcotrione	2-(2-Chloro-4-(methylsulfonyl)benzoyl)-1,3-cyclohexanedione	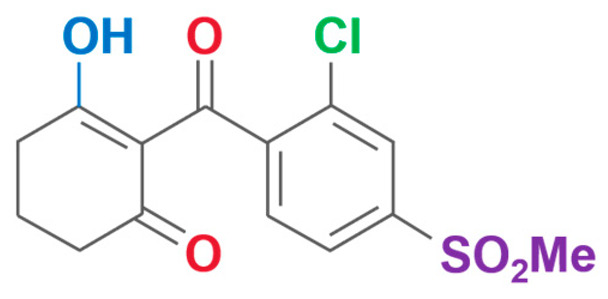	JLK Pharmaceutical Technology Co., Ltd (Beijing, China)	99105-77-8
Tefuryltrione	2-{2-chloro-4-(methylsulfonyl)-3-[(tetrahydrofuran-2-ylmethoxy)methyl]benzoyl}cyclohexane-1,3-dione	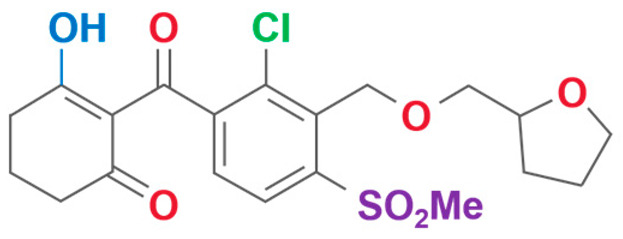	Bayer CropScience Co., Ltd (Hangzhou, China)	473278-76-1
Benzobicyclon	3-[2-chloro-4-(methylsulfonyl)benzoyl]-4-(phenylthio)bicyclo[3.2.1]oct-3-en-2-one	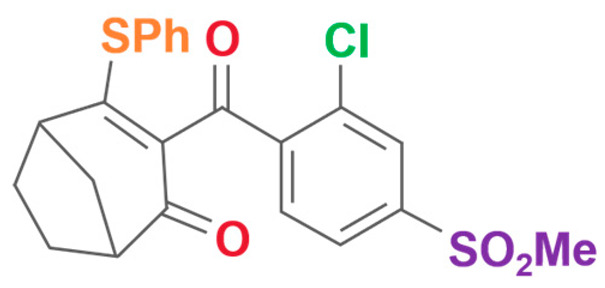	One Plus One Biotechnology Co., Ltd (Wuhan, China)	156963-66-5
Mesotrione	2-(4-Methylsulfonyl-2-nitrobenzoyl)-1,3-cyclohexanedione	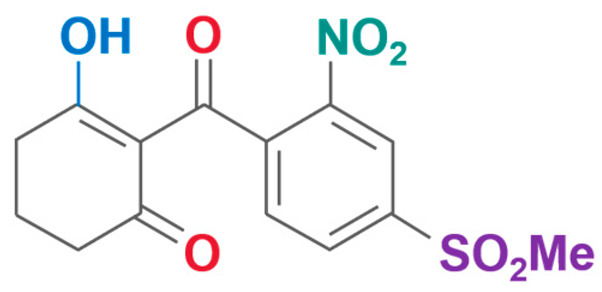	Xianda Agrochemical Co., Ltd (Binzhou, China)	104206-82-8
Tembotrione	2-{2-chloro-4-mesyl-3-[(2,2,2-trifluoroethoxy)methyl]benzoyl}cyclohexane-1,3-dione	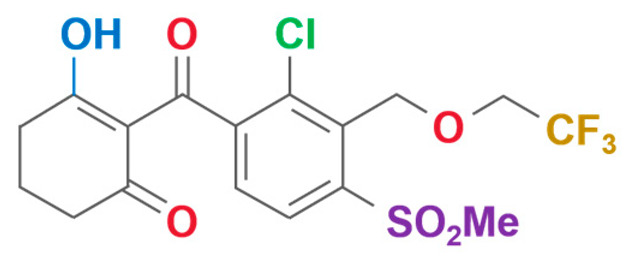	Bayer CropScience Co., Ltd (Hangzhou, China)	335104-84-2
Bicyclopyrone	4-Hydroxy-3-{2-[(2-methoxyethoxy)methyl]-6-(trifluoromethyl)-3-pyridinylcarbonyl}bicyclo[3.2.1]oct-3-en-2-one	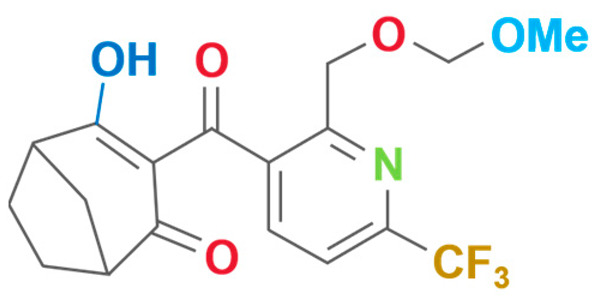	Huisheng Agricultural Technology Co., Ltd (Beijing, China)	352010-68-5

## Data Availability

Data will be made available on request.

## Supplementary Materials

The following supporting information can be downloaded at: https://www.mdpi.com/article/10.3390/plants13131796/s1, Table S1: primer sequences; Table S2: coding sequences of 8 glycosyltransferase genes; Table S3: amino acid sequences of 8 glycosyltransferase genes; Table S4: expression of eight glycosyltransferase genes induced by triketone herbicides.
