# Supplementary material for: First Clarification of the Mechanism of Action of the Apple Glycosyltransferase MdUGT91AJ2 Involved in the Detoxification Metabolism of the Triketone Herbicide Sulcotrione
Source: Plants (Basel). 2024 Jun 28;13(13):1796. doi: 10.3390/plants13131796 (PMC11244407; doi:10.3390/plants13131796)
Supplement: Supplementary file 1 [file plants-13-01796-s001.zip › plants-3027311-supplementary.pdf]

Table S1 Primer sequences

Gene name	Primer sequence	Function
qMdUGT91AJ1	F: GCTCAAAAGGGTCACCAAATC<br>R: GGTAGCTCGGAACTCGGC	For Real-time PCR
qMdUGT91AJ2	F: TAGCATTGCAAAGGTGGGGTG<br>R: CGATGAGCGGGGAAAAGGAG	For Real-time PCR
qMdUGT91AJ3	F: ATGCACGAAAAGCAGGTTGG<br>R: CCCGTACTAACTCGCCCTCT	For Real-time PCR
qMdUGT91AJ4	F: CGAAAAGCAGGTTGGGTTGG<br>R: TTTGCCCGTACTAACTCGCC	For Real-time PCR
qMdUGT91AJ5	F: TCGCGGGTTCGATTCAAGAT<br>R:CATGCTGATGTGGTGCTGAG	For Real-time PCR
qMdUGT91AJ6	F: TGGCTCGAAAGTAAAGAAACCG<br>R: CCCAAATAAAGGGCAACCCG	For Real-time PCR
qMdUGT91AJ7	F: GGTTGGGGGTTTCCTTACCC<br>R: CTCCAACCCAACCCGCTTAT	For Real-time PCR
qMdUGT91C7	F: CTATGCCCCTCACTGGCTTC<br>R: GGACCGAGGTAGGCCAGG	For Real-time PCR
qMdUBQ	F: CGTGGTGGTTTTTAAG<br>R: GCAGAAACAGTACCAT	For Real-time PCR
cMdUGT91AJ2	F:GGATCCATGGAGAGCAAAGATCAG<br>R:GAGCTCTTAGGTTGAAGTGGGCC	For amplifying full-length cDNA

Table S2 Coding sequences of 8 glycosyltransferase genes

MdUGT91AJ1 (MD02G1083600) CDS sequences

ATGGAGAGCAAAGATCAGAAGAAACTTCACATAGCCATGTTCCCATGGCTAGCCTACG
GCCACCTAATGTCATTTCTGGAGGTCTCCAAGTTCTTAGCTCAAAAGGGTCACCAAATC
TCCTTCATCTCCACACCCAAAAACATCAATCGCTTCCGATCCTCCTCCCTTTCCCCTCTT
ATCGATTTCATCGAGCTTCCTCTCCCCGCCGTCGACGGCTTACCGGAATCCGCCGAGTT
CCGAGCTACCAATCAACAAATCACGCATTTCATCCAACATTCGGGCGTCAACTGGGTC
GTCCACGACGTCATTTGCTACTGGCTTCCCCGAGTTGCCACCCAGCTCGGAGTCAACT
CGGTATTCTTCAGCGTCACCAACGCCACTACCTTGGCCTTTTTCGGTCCTCCGTCCGAG
TTACTCAGCGACAAACTCCGGCGGCCAGAGGACTTTACGGTCGTCCCCGAGTGGGTCG
ACTATCCTTCCAACGTTGCTTTTAAGCTGCATGAGATGGTGAGCCACTGGGACTGCATG
GAGGACGAGGTCTCTGATTTTCAGAGAGCCGCGCGTGCGATTCAAGATTGTAATTTTGT
GACTATGCGGAGCTGCACCGAGTTTGAATCTGACGCGGTGAGTCTGCTCAAGAAGATC
TACGGCAAACCCATTGTTCCACTAGGGCTGCTGCCGCCCGACTCAGCGCCGCATCAGC
ATGGCGTCGCTGACGATCGATGGGATGAGAAGTGGGAAGTGTTGAGAGAGTGGCTCG
AAAATAAGAAACCGAACTCGGTGGTTTACATCGCATTCGGCACTGAGGTGACTCCTAG
CCAAGAGTTGATGCATGAGTTGGCACATGGGATAGAGAAATCCGGGTTGCCCTTTATTT
GGGTGGTCAACAATCGCCCGTTGGTGGAGGGTGTGTTGGGGGCCGATATCATTCCACC
CGGGTTTCAAACTCGAGTGGAGGATCGAGGCTTGGTCTGGAGGGGTTGGGCCCCACA
ACTTAAGATTCTGGATCATGCATCGGTCGGAGGTTTCCTAACTCATTGTGGTTGGAGTT
CAGTTGTTGAGGCACTACGGTTTGGGCTGGTTCTGATTTTGTTTTCAGGTGCTTATTCA
GACCAACCGTTGAACGCGAGACTAATGCACGATAAGCGGGTGGGGTTGGAGATACCA
AGGGACGAGCGAGACGGATCGTTTACGAGTGACTCGGTGGCCGAGTTGATTAGGCGA
GTGATGGTGGAGAAAGAAGGTGAGTCAATAAGGTCAAATGCATGGGCCATGAAGGAG
ATCTTTGGCAACGTAGAGTTGAACAAGCAGTGCTTCGACGAGTTTTCTCGGGTCCTTG
AAACCTGGCCAATTCCACCTAGTCAATGA

MdUGT91AJ2 (MD02G1084000) CDS sequences

ATGGAGAGCAAAGATCAGAAGAAACTTCACATAGCCATGTTCCCATGGCTAGCATTG
CAAAGGTGGGGTGCCTACGGCCACCTAATGTCATTTCTCGAGGTCTCCAAGTTCTTAG

CTCAAAAGGGTCATCGAATCTCCTTCATCTCCACCCCCAAAAACATCAATCGTCTCCG<br>ATCCTCCTCCTTTTCCCCGCTCATCGATTTCGTCGAGCTTCCTCTCCCTGCCGTCGACG<br>GCTTACCGGAATCCGCCGAGTCCACCTCCGAGCTACCAATCAACAAAGTCCCATACCT<br>CAAAAAAGCATACGACTTGCTCAAGCCTTCAGTCACCCATTTCATCGAACACTCGGGC<br>GTCAACTGGATCGTCCACGACTTCATTTGCTACTGGGTTCCCCGAGTCGCCACTCAGC<br>TCGGAGTCAACTCGGTCTACTTCAGCGTCACCAACGCCAATTCCTTGGTTTTCATCGG<br>TTCTCCGTCCGAGTTACTCAGCAATAAACGCAGGCGGCCGGAGGACTTTACTGTCGTC<br>CCCGAGTGGGTCGACTATCCTTCCAACGTTGCTTTTAAACTGCATGAGATGGTGACCC<br>ACTGGGATTGCATGGACGACGAGGTCTCCGATTTTCAGAGACTCGCAGGTGCGATTC<br>AAGATTGTAATTTTGTTACTATGCGGAGCTGCACCGAGTTCGAATCTGACGCTGTAAG<br>TCTGCTCAGGAAGATCTACGGCAAACCAGTTGTTCCACTCGGGATGCTGCCGCCAGTC<br>TCAGCGCCGCATCAGCACGGCGTCGCTGATGATCGAGGGGATGAGACGTGGGAAGTG<br>CTGAGAGGTTGGCTCGAAAATAAGAAACCGAACTCGGTGGTTTACATCGCATTCGGC<br>ACTGAGGTGACTCCGAGTCTAGAATTGATGCACGAGTTGGCACATGGGATAGAGAAA<br>TCCGGGTTGCCCTTTATCTGGGTGGTCAACAATCGCCCCTTAGTGGAGGGTGTGTTGG<br>GGGCTGATATCATTCCACCCGGGTTTCAAACTCGAGTGGAGGATCGAGGCTTGGTCTG<br>GAGGGGTTGGGCCCCACAACTTAAGATTCTAGCTCATGCATCGATCGGAGGTTTCCTA<br>ACTCATTGCGGTTGGAGTTCAGTTGTTGAGGCACTACGGTTTGGGCGGGTTTTGATTT<br>TGTTTTCGGGTGCTTATTCAGACCAACCGTTGAACGCAAGACTAATGCATGATAAGCG<br>GGTCGGGTTGGAGATACCGAGGGACGAGCAAGACGGATCGTTTACGAGTGACTCGGT<br>GGCGGAGTTGATTAGGCGAGTGATGGTGGAGAAAGAAGGTGAGTCAATAAGGTCAA<br>ATGCATGGGCCATGAAGGAGATCTTTGGCAACGTAGAGTTGAACAACAGGTGCTTGG<br>ACGAATTCACTCGAACCCTTGAAACCTGGCCCACTTCAACCTAA
**MdUGT91AJ3 (MD15G1211400) CDS sequences**<br>ATGGAGCGCAAAGATGAGAAGAAACTTCACATCGCCATGTTCCCATGGCTAGCCTACG<br>GCCACCTGATGCCATTTCTCGAGGTCTCCAAGTTCTTAGCTCAAAAAGGTCACCAAATC<br>TCCTTCATCTCCACCCCCCAAAACATCAATCGCCTCCGGTCATCCTCCCTTTCCCTACTC<br>ATCGATTTCGTTGAGCTTCCTCTCCCCGCCGTCGACGGCTTACCTGAATCCGTCGAGTC<br>CACCTCCCAGCTACCAATCAACAAAGTCCCCTACCTCAAAAAAGCCTACGACTTGCTC

AAGCCTGCAGTCACGCATTTCGTCCAACACTCGGGCGTCAACTGGGTCGTCCACGACG
TTATTTGCTACTGGCTGCCCCAAGTCGCCACTCAGCTCGGAGTCAACTCGGTCTTCTTC
AGCATCATCAACGCCACTTCCTTAGCTTTCGGCGGTCCTCCGTCTGAGTTACTCGGCGA
TAAACGCCGGCGGCCGGAGGACTTTACGGTCGTTCCCGAGTGGTTCGACTATCCTTCC
ACCGTCGCTTATAAGCTGCACGAGATGGTGAGTCATTGGGATTGCATGGACGACAATGT
CTCCGATTTTCAGAGGCTCGCGGTTACGGTTCAAGATTGTAATTTTGTGACTATACGGA
GCTGCACCGAGGTTGAATCTGACGCGGTGAGTCTGCTCAGAAATTTATACGGTAAACC
CGTTGTTCCACTCGGGCTGCTGCCGACCAGCTCAGCACCGCATCAGCCCGGCGGCACT
AACGATGGAGCGGATGAGAAGTGGGAAGTGTTGAGAGAGTGGCTCGAAAATAAGAAA
CCGAACTCGGTGGTTTACATCGCACTCGGCACCGAGGTGACTCTGAGTCAAGAACTTA
TGCACGAGTTGGCTCACGGGATAGAGAAATCGGGGTTGCCCTTTATTTGGGTGGTCAA
CAATCGCCCGTTAGTGGAGGACGCGTTGGGGTCTAATATCCTTCCACTCGGATTTGAAA
CCCGAGTGGACGGTCGGGGCTTTGTGTGGAGGGGTTGGGCCCCACAACGTAAGATTTT
GGGTCATATCTCAGTTGGAGGATTCCTGACTCATTGCGGTTGGAGTTCAATTATTGAAG
CATTAGGGTTTGGGAGGGTTTTGATTTTGTTTTCGGGTGCAAATTCGGACCAAGGGTTG
AACTCGAGACTAATGCACGAAAAGCAGGTTGGGTTGGAGATACCGAGGGACGAGCGA
GATGGGTCGTTTACGAGTGACTCGGTGGCCGAGTTGATTAGGCGAGTGATGGTGGAGA
AAGAGGGCGAGTTAGTACGGGCAAATGCACGGGCCATGAAGGAGATATTTGGCAACGT
AGAGTTGAACAAAAAGTGCTTGGACGAGTTCACTCGGGTCCTTGAAACTTGGCCCGC
TTCAACTTAA

MdUGT91AJ4 (MD10G1101200) CDS sequences

ATGGAGCGCAAAGATGAGAAGAAACTTCACATCGCCATGTTCCCATGGCTAGCCTACG
GCCACCTGATGCCATTTCTCGAGGTCTCCAAGTTCTTAGCTCAGAAAGGTCACCAAATC
TCCTTCATCTCCACCCCCCAAAATATCAATCGCCTCCGGTCATCCTCCCTTTCCCTACTC
ATCGATTTCGTTGAGCTTCCTCTCCCCGCCGTCGACGGCTTACCTGAATCCGTCGAGTC
CACCTCCCAGCTACCAATCAACAAAGTCCCCTACCTCAAAAAAGCCTACGAATTGCTC
AAGCCTGCAGTCACGCATTTCGTCCAACACTCGGGCGTCAACTGGGTCGTCCACGACG
TTATTTGCTACTGGCTGCCCGAAGTCGCCACTCAGCTCGGAGTCAACTCGGTCTTCTTC
AGCATCATCAACGCCACTTCCTTAGCCTTCGGCGGTCCTCCGTCTGAGTTACTCGGTGA

TAAACGCCGGCGGCCGGAGGACTTTACGGTCGTTCCCGAGTGGTTCGACTATCCTTCC
ACCGTCGCTTATAAGCTGCACGAGATGGTGAGTCATTGGGATTGCATGGACGACAATGT
CTCCGATTTTCAGAGGCTCGCGGTTACAGTTCAAGATTGTAATTTTGTGACTATACGGA
GCTGCACCGAGGTTGAATCTGACGCGGTGAGTCTGCTCAGAAATTTATACGGCAAACC
CGTTGTTCCACTCGGGCTGCTGCCGACCAGCTCAGCACCGCATCAGCCCAGCGGCGCT
AACGATGGAGCGGATGAGAAGTGGGAAGTGTTGAGAGAGTGGCTCGAAAATAAGAAA
CCGAACTCGGTGGTTTACATCGCACTCGGCACCGAGGTGACTCTGAGTCAAGAGCTTA
TGCACGAGTTGGCTCACGGGATAGAGAAATCGGGGTTGCCCTTTATTTGGGTGGTCAA
CAATCGCCCGTTAGTGGAGGGCGCGTTGGGGTCTAATATCCTTCCACTCGGATTTGAAA
CCCGAGCGGACGGTCAGGGCTTTGTGTGGAGGGGTTGGGCCCCACAACGTAAGATTTT
GGGTCATATCTCAGTTGGAGGATTCCTGACTCATTGCGGTTGGAGTTCAATTATTGAAG
CATTAGGGTTTGGGAGGGTTTTGATTTTGTTTTCAGGTGCAAATTCGGACCAAGGGTTG
AACGCGAGACTAATGCACGAAAAGCAGGTTGGGTTGGAGATACCGAGGGACGAGCGA
GATGGGTCGTTTACGAGTGACTCGGTGGCCGAGTTGATTAGGCAAGTGATGGTGGAGA
AAGAGGGCGAGTTAGTACGGGCAAATGCATGGGCCATGAAGGAGATATTTGGCAACGT
AGAGTTGAACAAAAAGTGCTTGGACGAGTTCACTCGGGTCCTTGAAACTTGGCCCGC
TTCAACTTAA

MdUGT91AJ5 (MD02G1083100) CDS sequences

ATGCCATTTCTGGAGGTCTCCAAGTTCTTAGCTCAAAAGGGTCACCAAATCTCTTTCAT
CTCCACCCCCAAAAACATCAATCGCCTCCGATCCTCCTCCTTTTCCCCGCTCATCAATTT
CGTCGAGCTTCCTCTCCCCGCCGTCGACGGCTTACCGGAATCCGTTGAGTCCACCTCC
GAGCTACCAATCAACAAAGTCCCTTACCTCAAAAAAGCATACGACTTGCTTAAGCCTT
CAGTCACGCATTTCATCCAACACTCGAGCGTCAACTGGGTCGTCCATGACATCATTTGC
TACTGGATGCCCAGAGTCGCCACTCAGCTGGGAGTCAACTCGGTCTACTTCAACATCA
CCAACGCCAGTACCTTGGCTTTCTTGGGTCCTCCGACCGAGTTACTCGGCGATAAACG
CAGGCGACCGGAGGACTTTACGGTCGTCCCCGAGTGGGTCGACTATCCTTCCAACGTT
GCTTTTAAGCAGCATGAGATGGTGAGCCACTGGGATTGCATGGACGATGAGGTCTCCG
ATTTTCAGAGACTCGCGGGTTCGATTCAAGATTGTAATTTTGTGACTATGCGGAGCTGC
ACCGAGTTCGAATCTGACGCGGTGAGTTTGCTCAGGAAGATCTACGGCAAACCCGTTG

TTCCACTCGGGCTGCTTCCGCCTGGCTCAGCACCACATCAGCATGGCGTCGCTAACGAT
CGAGGGGATGACAAGTGGGAAGTGTTGAGAGAGTGGCTCGAAAGTAAGAAACCGAA
CTCGGTGGTTTACATCGCCCTCGGCACCGAGGTGACTCTGAGTCAAGAGTTGATGCAC
GAGTTGGCACATGGGATAGAGAAATCCGGGTTGCCCTTTATTTGGGTGGTCAACAATC
GCCCGTTGGTGGAGGGCGTGTTGGGCTCCGATATAATTCCACTCGGGTTTGAAACACG
AGTAGAGGATCGAGGCTTGGTCTTGAGAGGTTGGGCCCCACAACTTAAGATTCTAGGT
CATATTTCGATCGGAGGTTTCCTAACTCATTGTGGTTGGAGTTCAGTTGTCGAGGCACT
AGGGTACGGGCGGGCTTTGATTTTGTTTTCGGGTGCTAATTCCGACCAAGGGTTGATCG
CGAGACTAATGCACGATAAGCAGGTCGGGTTGGAGATACCGAGGGACGAGCAAGACG
GGTCGTTTACGAGTGACTCGGTGGCCGAGTTGATTGGGCGAGTGATGGTGGAGAAAG
AAGGTGAGTCCATAAGGTCAAATGCACGGGCCATGAAGGAGATATTTGGCAATGTAGA
GTTGAACAACAAGTGCTTGGACGAGTTCACTCGGGTCCTTGAAACCTGGCCCAAATAA

MdUGT91AJ6 (MD02G1083300) CDS sequences

ATGGAGAGCAAAGATCAGAAGAAACTTCACATAGCCATGTTCCCATGGCTAGCCTACG
GACACCTAATGCCATTTCTGGAGGTCTCCAAGTTCTTAGCTCAAAAGGGTCACCAAAT
CTCTTTCATCTCCACCCCCAAAAACATCAATCGCCTCCGATCCTCCTCCTTTTCCCCGCT
CATCAATTTCGTCGAGCTTCCTCTCCCCGCCGTCGACGGCTTACCGGAATCCGTTGAGT
CCACCTCCGAGCTACCAATCAACAAAGTCCCTTACCTCAAAAAAGCATACGACTTGCT
CAAGCCTTCAGTCATGCATTTCATCCAACACTCGAGCGTCAACTGGGTCGTCCATGACA
TCATTTGCTACTGGATGCCCAGAGTCGCCACTCAGCTGGGAGTCAACTCGGTCTACTTC
AACATCACCAACGCCAGTACCTTGGCTTTCTTGGGTCCTCCGACCGAGTTACTCGGCG
ATAAACGCAGGCGACCGGAGGACTTTACGGTCGTCCCCGAGTGGGTCGACTATCCTTC
CAACGTTGCTTTTAAGCAGCATGAGATGGTGAGCCACTGGGATTGCATGGACGATGAG
GTCTCCGATTTTCAGAGACTCGCGGGTTCGATTCAAGATTGTAATTTTGTGACTATGCG
GAGCTGCACCGAGTTCGAATCTGACGCGGTGAGTTTGCTCAGGAAGATCTACGGCAAA
CCCGTTGTTCCACTCGGGCTGCTTCCGCCTGGCTCAGCACCACATCAGCATGGCGTCG
CTAACGATCGAGGGGATGACAAGTGGGAAGTGTTGAGAGAGTGGCTCGAAAGTAAAG
AAACCGAACTCGGTGGTTTACATCGCCCTCGGCACCGAGAGAAATCCGGGTTGCCCTT
TATTTGGGTGGTCAACAATCGCCCGTTGGTGGAGGGCGTGTTGGGCTCCGATATAATTC

CACTCGGGTTTGAAACACGAGTAGAGGATCGAGGCTTGGTCTTGAGAGGTTGGGCCC
CACAACTTAAGATTCTAGGTCATATTTCGATCGGAGGTTTCCTAACTCATTGTGGTTGGA
GTTCAGTTGTCGAGGCACTAGGGTACGGGCGGGCTTTGATTTTGTTTTCGGGTGCTAAT
TCCGACCAAGGGTTGATCGCGAGACTAATGCACGATAAGCAGGTCGGGTTGGAGATAC
CGAGGGACGAGCAAGACGGGTCGTTTACGAGTGACTCGGTGGCCGAGTTGATTGGGC
GAGTGATGGTGGAGAAAGAAGGTGAGTCCATAAGGTCAAATGCACGGGCCATGAAGG
AGATATTTGGCAATGTAGAGTTGAACAACAACAAGTGCTTGGACGAGTTCACTCGGGT
CCTTGAAACCTGGCCCAAATAA

MdUGT91AJ7 (MD02G1083800) CDS sequences

ATGGAAAGCAAAGATCAGAAGAAACTTCAAATAGCCATGTTCCCATGGCTAGCCTACG
GCCACCTAATGCCATTTCTCGAGGTCTCCAAGTTCTTGGCTCAAAAGGGTCACCAAATC
TCCTTCATCTCCACCCCCAAAAACATCAATCGCCTCCGATCCTCCTCCTTTTCCCCGCTC
ATCGATTTCGTCGAGCTTCCTCTCCCCGCTGTCGACGGCTTACCGGAATCCGTCGAGTC
CACCTCCGAGCTACCAATCAACAAAGTCCCTTACCTCAAAAAAGCATACGACTTGCTC
AAGCCTTCAGTCATGCATTTCATCCAGCACTCGGGAGTCAACTGGGTCGTCCATGACTT
CATTTGCTACTGGATGCCCAGGGTCGCCACTCAGCTGGGAGTCAACTCGGTCTACTTC
AACATCACCAACGCCACTACCTTGGCTTTCTTCGGTCCTCCGTCCGAGTTACTCGGCAA
TAAACGCAGGGGGCCGGAGGACTTTACGGTCGTCCCCGAGTGGGTCGACTATCCTTCC
AACGTTGCTTTTAAATTGCATGAGATGGTGACCCACTGGGATTGCATGGGCGACGAGG
TCTCCGATTTTCAGAGACTCGGGCGTACGATTCAAGATTGTAATTTTGTGACTATGCGG
AGCTGCACCGAGTTCGAATCTGACGCAGTGAGTCTGCTCAGGAAGATCTACGGCAAAC
CAGTTGTTCCACTCGGGCTGCTGCCGCCGGTCTCAGCGCCGCAACAGCACGGCGTCGC
TGACGATCGAGGGGATGAGACGTGGGAAGTGCTGAGAGGTTGGCTCGAAAATAAGAA
ACCGAACTCGGTGGTTTACATCGCCCTCGGCACCGAGGTGACTCTGAGTCAAGAGTTG
ATGCACGAGTTGGCACATGGGATGGAGAAATCCGGGTTGCCTTTTATTTGGGTGGTCA
ACAATCGCCCGTTAGTGGAAGGCTTGTTGGGCTCCGATATAATTCCACTCGGATTTGAA
ACCCGAGTAGAGAATCGAGGCTTGGTCTTGAGAGGTTGGGCTCCACAACTTAATATTCT
AGGTCATATCTCGGTTGGGGGTTTCCTTACCCATTGTGGTTGGAGTTCAGTTGTCGAGG
CACTAGGGTACGGGCGGGCTTTGATTTTGTTTTCAGGTGCTAATTCAGACCAAGGGTTA

ATCTCTAGACTAATGCACGATAAGCGGGTTGGGTTGGAGATACCGAGGGACGAGCAAG
ATGGGTCGTTTACGAGTGACTCGGTGGCCGAGTTGACAAGGCGAGTGATGGTGGAGA
AAGAAGGTGAGTCCATAAGGTCAAATGCATGGGCCATGAAGGAGATATTTGGCAACGT
AGAGTTGAACAACAAGTGCTTGGATGAGTTCACTGGGGTCCTTGAAACTTGGCCCACT
TCCTCCTAG

MdUGT91C7 (MD08G1200700) CDS sequences (Variable shear)

TGGACACAGGTCAATCTCCATGGACAACCAGAACACTCAGAGTGGAGGCCAAGGAGG
AGGAGGCGGAGGAGCTGTTCTTCACGTGGTGGTGTTTCCATGGCTGGCCATGGGCCAC
CTCATCCCCTTCTTCCACCTTTCCACCCTCATAGCTCAAAGGGGTCACACCGTCTCCTT
CGTCTCCACCCCAAGGAACCTCTCCAGACTTCCCAAAATACCCTCCCACCTCTCCTCCC
TCGTCAACCTCGTCTCCTTCCCTCTCCCCCGCCTCCCTAACCTCCCAAACGACGCCGAA
TCATCAACCGACGTCCCCTTCCACAAGCAGCAACTACTCAAAACGGCCTTCGACATGC
TACAAACCCCGCTGACCGCTTTCCTGGAGTCCTCAAGACCCGACTGGGTTATTTACGA
CTATGCCCCTCACTGGCTTCCCGCCATAGCCGCCAGGCTCGGCGTCGCACACGCCTTCT
TTTTCTGTGTCAACGCCGCCTGCCTGGCCTACCTCGGTCCGCCGTCGGTTCTGATCAGC
GGCCAGGATGGGAGGACCAAGGCCGAGGATTTCACGGTGGTCCCCAAGTGGGTCCCG
TTCGAGTCCGACATGGCATATCGGCTCCACGAGGTTGCCAAGTGGGTGCAAGCATCAA
GCGGGAACGAGTCGGGCACTCCCGATACGGTGCGTTTTGGGGTTGCGATTGAGGAGA
GCGACGTTGTGTTTGTTAGAAGCTCTGACGAGTTTGAGCCCGAGTGGTTAAATTTGGT
GAGAGAGCTTTACCGCGAGGTAAAAGTAAAACCCGTTGTTCCGGTTGGATTTTTACCG
CCTAACATAGAAGAGGAGGCAAGTGAATTTGATGAAACATGGGGTGGCATTAAAGGGT
GGTTGGACAAGCAACGAGTCAACTCGGTGGTTTACATTGCACTCGGGACCGAAGCGA
CACTGAGTCAGGAGGAACTCACCGAGCTGGCTCTCGGGTTGGAGCTGTCCGGGGTAC
CCTTCTTTTGGGTGTTGAGAAACCCGCCCGAGTCGACTCAGTCAGTGTCTGAGATGCT
TCCTCCAGGGTTTTTGGAACGAGTCAAGGGTCGAGGTGTGGTGGACTTGGGGTGGGC
TCCGCAGGTGCGCATACTGAGTCATGACTCGGTGGGGGGATTCTTGACTCACTGCGGT
TGGAACTCAATGATCGAAGGGCTCATGTTCGGACGGGTTTTGATGTTTTTCCGATGGT
GAACGACCAAGGGCTTAATGCTCGATTGGGGAATGGAAAGGGGCTCGGGGTGGAAAT
ACCTAGGAACGAACGAGATGGGTCGTTTACTCGTGACTCGGTGGCTGAGTTTGTAAGG

TTGGCAATGGTGGACGACTCGGGTGAATCGATGAGGATAAGGGCCAAGGAAATGAAG GATTTGTTTGGAGACAGAAATAAGAACAATCGAATAGTGGGTGAATTCATATGTTTTCT CGAAGAGAACAGGCCACCGAGGTGTCCAGAATAA

Table S3 Amino acid sequences of 8 glycosyltransferase genes

**MdUGT91AJ1 (MD02G1083600) amino acid sequences**<br>MESKDQKKLHIAMFPWLAYGHLMSFLEVSKFLAQKGHQISFISTPKNINRFRSSSLSPLIDF IELPLPAVDGLPESAEFRATNQQITHFIQHSGVNWVVHDVICYWLPRVATQLGVNSVFFS VTNATTLAFFGPPSELLSDKLRRPEDFTVVPEWVDYPSNVAFKLHEMVSHWDCMEDEVS DFQRAARAIQDCNFVTMRSCTEFESDAVSLLKKIYGKPIVPLGLLPPDSAPHQHGVADDR WDEKWEVLREWLENKKPNSVVYIAFGTEVTPSQELMHELAHGIEKSGLPFIWVVNNRPL VEGVLGADIIPPGFQTRVEDRGLVWRGWAPQLKILDHASVGGFLTHCGWSSVVEALRFG LVLILFSGAYSDQPLNARLMHDKRVGLEIPRDERDGSFTSDSVAELIRRVMVEKEGESIRS NAWAMKEIFGNVELNKQCFDEFSRVLETWPIPPSQ
**MdUGT91AJ2 (MD02G1084000) amino acid sequences**<br>MESKDQKKLHIAMFPWLALQRWGAYGHLMSFLEVSKFLAQKGHRISFISTPKNINRLRSS SFSPLIDFVELPLPAVDGLPESAESTSELPINKVPYLKKAYDLLKPSVTHFIEHSGVNWIVHD FICYWVPRVATQLGVNSVYFSVTNANSLVFIGSPSELLSNKRRRPEDFTVVPEWVDYPSNV AFKLHEMVTHWDCMDDEVSDFQRLAGAIQDCNFVTMRSCTEFESDAVSLLRKIYGKPVV PLGMLPPVSAPHQHGVADDRGDETWEVLRGWLENKKPNSVVYIAFGTEVTPSLELMHEL AHGIEKSGLPFIWVVNNRPLVEGVLGADIIPPGFQTRVEDRGLVWRGWAPQLKILAHASIG GFLTHCGWSSVVEALRFGRVLILFSGAYSDQPLNARLMHDKRVGLEIPRDEQDGSFTSDSV

AELIRRVMVEKEGESIRSNAWAMKEIFGNVELNNRCLDEFTRTLETWPTST
**MdUGT91AJ3 (MD15G1211400) amino acid sequences**<br>MERKDEKKLHIAMFPWLAYGHLMPFLEVSKFLAQKGHQISFISTPQNINRLRSSSLSLLIDF<br>VELPLPAVDGLPESVESTSQLPINKVPYLKKAYDLLKPAVTHFVQHSGVNWVVHDVICY<br>WLPQVATQLGVNSVFFSIINATSLAFGGPPSELLGDKRRRPEDFTVVPEWFDYPSTVAYKL<br>HEMVSHWDCMDDNVSDFQRLAVTVQDCNFVTIRSCTEVESDAVSLLRNLYGKPVVPLG<br>LLPTSSAPHQPGGTNDGADEKWEVLREWLENKKPNSVVYIALGTEVTLSQELMHELAHG<br>IEKSGLPFIWVVNNRPLVEDALGSNILPLGFETRVDGRGFVWRGWAPQRKILGHISVGGFL<br>THCGWSSIIEALGFGRVLILFSGANSDQGLNSRLMHEKQVGLEIPRDERDGSFTSDSVAELI<br>RRVMVEKEGELVRANARAMKEIFGNVELNKKCLDEFTRVLETWPAST
**MdUGT91AJ4 (MD10G1101200) amino acid sequences**<br>MERKDEKKLHIAMFPWLAYGHLMPFLEVSKFLAQKGHQISFISTPQNINRLRSSSLSLLIDF<br>VELPLPAVDGLPESVESTSQLPINKVPYLKKAYELLKPAVTHFVQHSGVNWVVHDVICY<br>WLPEVATQLGVNSVFFSIINATSLAFGGPPSELLGDKRRRPEDFTVVPEWFDYPSTVAYKL<br>HEMVSHWDCMDDNVSDFQRLAVTVQDCNFVTIRSCTEVESDAVSLLRNLYGKPVVPLG<br>LLPTSSAPHQPSGANDGADEKWEVLREWLENKKPNSVVYIALGTEVTLSQELMHELAHG<br>IEKSGLPFIWVVNNRPLVEGALGSNILPLGFETRADGQGFVWRGWAPQRKILGHISVGGFL<br>THCGWSSIIEALGFGRVLILFSGANSDQGLNARLMHEKQVGLEIPRDERDGSFTSDSVAE<br>LIRQVMVEKEGELVRANAWAMKEIFGNVELNKKCLDEFTRVLETWPAST
**MdUGT91AJ5 (MD02G1083100) amino acid sequences**<br>MPFLEVSKFLAQKGHQISFISTPKNINRLRSSSFSPLINFVELPLPAVDGLPESVESTSELPINK<br>VPYLKKAYDLLKPSVTHFIQHSSVNWVVHDIICYWMPRVATQLGVNSVYFNITNASTLAF<br>LGPPTELLGDKRRRPEDFTVVPEWVDYPSNVAFKQHEMVSHWDCMDDEVSDFQRLAGSI<br>QDCNFVTMRSCTEFESDAVSLLRKIYGKPVVPLGLLPPGSAPHQHGVANDRGDDKWEVL<br>REWLESKKPNSVVYIALGTEVTLSQELMHELAHGIEKSGLPFIWVVNNRPLVEGVLGSDII<br>PLGFETRVEDRGLVLRGWAPQLKILGHISIGGFLTHCGWSSVVEALGYGRALILFSGANSD<br>QGLIARLMHDKQVGLEIPRDEQDGSFTSDSVAELIGRVMVEKEGESIRSNARAMKEIFGNV<br>ELNNKCLDEFTRVLETWPK
**MdUGT91AJ6 (MD02G1083300) amino acid sequences**

MESKDQKKLHIAMFPWLAYGHLMPFLEVSKFLAQKGHQISFISTPKNINRLRSSSFSPLINF
VELPLPAVDGLPESVESTSELPINKVPYLKKAYDLLKPSVMHFIQHSSVNWVVHDIICYW
MPRVATQLGVNSVYFNITNASTLAFLGPPTELLGDKRRRPEDFTVVPEWVDYPSNVAFKQ
HEMVSHWDCMDDEVSDFQRLAGSIQDCNFVTMRSCTEFESDAVSLLRKIYGKPVVPLGL
LPPGSAPHQHGVANDRGDDKWEVLREWLESKETELGGLHRPRHREKSGLPFIWVVNNRP
LVEGVLGSDIIPLGFETRVEDRGLVLRGWAPQLKILGHISIGGFLTHCGWSSVVEALGYGR
ALILFSGANSDQGLIARLMHDKQVGLEIPRDEQDGSFTSDSVAELIGRVMVEKEGESIRSN
ARAMKEIFGNVELNNNKCLDEFTRVLETWPK

MdUGT91AJ7 (MD02G1083800) amino acid sequences

MESKDQKKLQIAMFPWLAYGHLMPFLEVSKFLAQKGHQISFISTPKNINRLRSSSFSPLIDF
VELPLPAVDGLPESVESTSELPINKVPYLKKAYDLLKPSVMHFIQHSGVNWVVHDFICYW
MPRVATQLGVNSVYFNITNATTLAFFGPPSELLGNKRRGPEDFTVVPEWVDYPSNVAFKL
HEMVTHWDCMGDEVSDFQRLGRTIQDCNFVTMRSCTEFESDAVSLLRKIYGKPVVPLGL
LPPVSAPQQHGVADDRGDETWEVLRGWLENKKPNSVVYIALGTEVTLSQELMHELAHG
MEKSGLPFIWVVNNRPLVEGLLGSDIIPLGFETRVENRGLVLRGWAPQLNILGHISVGGFL
THCGWSSVVEALGYGRALILFSGANSDQGLISRLMHDKRVGLEIPRDEQDGSFTSDSVAE
LTRRVMVEKEGESIRSNAWAMKEIFGNVELNNKCLDEFTGVLETWPTSS

MdUGT91C7 (MD08G1200700) amino acid sequences

MDNQNTQSGGQGGGGGGAVLHVVVFPWLAMGHLIPFFHLSTLIAQRGHTVSFVSTPRNL
SRLPKIPSHLSSLVNLVSFPLPRLPNLPNDAESSTDVPFHKQQLLKTAFDMLQTPLTAFLESS
RPDWVIYDYAPHWLPAIAARLGVAHAFFFCVNAACLAYLGPPSVLISGQDGRTKAEDFTV
VPKWVPFESDMAYRLHEVAKWVQASSGNESGTPDTVRFGVAIEESDVVFVRSSDEFEPEW
LNLVRELYREVKVKPVVPVGFLPPNIEEEASEFDETWGGIKGWLDKQRVNSVVYIALGTE
ATLSQEELTELALGLELSGVPFFWVLRNPPESTQSVSEMLPPGFLERVKGRGVVDLGWAP
QVRILSHDSVGGFLTHCGWNSMIEGLMFGRVLMFFPMVNDQGLNARLGNGKGLGVEIPR
NERDGSFTRDSVAEFVRLAMVDDSGESMRIRAKEMKDLFGDRNKNNRIVGEFICFLEENR
PPRCPE

Table S4 Expression of eight glycosyltransferase genes induced by triketone herbicides

Compounds	Gene name	0 h	3 h	6 h	12 h	24 h
Sulcotrione	MdUGT91AJ1	1.00 ± 0.103	3.857 ± 0.264*	5.794 ± 0.325*	7.274 ± 0.293**	3.281 ± 0.227*
	MdUGT91AJ2	1.00 ± 0.112	8.394 ± 0.362**	14.365 ± 0.478**	18.298 ± 0.424**	10.466 ± 0.368**
	MdUGT91AJ3	1.00 ± 0.101	2.587 ± 0.193	4.487 ± 0.256*	3.265 ± 0.231*	2.108 ± 0.172
	MdUGT91AJ4	1.00 ± 0.098	2.019 ± 0.282	3.584 ± 0.261*	5.938 ± 0.365*	3.377 ± 0.194*
	MdUGT91AJ5	1.00 ± 0.106	2.476 ± 0.251	4.268 ± 0.228*	5.986 ± 0.236*	3.265 ± 0.281*
	MdUGT91AJ6	1.00 ± 0.114	2.108 ± 0.279	2.456 ± 0.196	1.945 ± 0.213	1.746 ± 0.225
	MdUGT91AJ7	1.00 ± 0.096	2.498 ± 0.202	3.157 ± 0.246*	4.052 ± 0.282*	2.575 ± 0.291
	MdUGT91C7	1.00 ± 0.094	2.174 ± 0.187	3.583 ± 0.191*	3.205 ± 0.225*	2.194 ± 0.212
Tefuryltrione	MdUGT91AJ1	1.00 ± 0.102	1.472 ± 0.192	1.928 ± 0.146	2.573 ± 0.157	2.043 ± 0.138
	MdUGT91AJ2	1.00 ± 0.111	1.284 ± 0.119	2.147 ± 0.125	2.943 ± 0.128	2.139 ± 0.112
	MdUGT91AJ3	1.00 ± 0.107	2.584 ± 0.127	3.579 ± 0.134*	4.978 ± 0.137*	3.295 ± 0.215*
	MdUGT91AJ4	1.00 ± 0.105	2.183 ± 0.176	3.466 ± 0.182*	2.955 ± 0.196	2.176 ± 0.154
	MdUGT91AJ5	1.00 ± 0.108	1.957 ± 0.181	2.461 ± 0.195	3.284 ± 0.213*	2.182 ± 0.207
	MdUGT91AJ6	1.00 ± 0.091	2.205 ± 0.156	2.748 ± 0.162	3.757 ± 0.171*	2.584 ± 0.198
	MdUGT91AJ7	1.00 ± 0.121	2.056 ± 0.148	2.756 ± 0.183	3.283 ± 0.168*	2.164 ± 0.183
	MdUGT91C7	1.00 ± 0.117	1.942 ± 0.157	2.651 ± 0.164	2.164 ± 0.181	1.478 ± 0.195
Benzobicyclon	MdUGT91AJ1	1.00 ± 0.105	1.947 ± 0.134	2.577 ± 0.147	3.295 ± 0.128*	2.176 ± 0.141
	MdUGT91AJ2	1.00 ± 0.108	1.473 ± 0.152	2.094 ± 0.168	3.574 ± 0.149*	2.573 ± 0.152
	MdUGT91AJ3	1.00 ± 0.113	1.855 ± 0.116	2.643 ± 0.175	3.958 ± 0.246*	2.461 ± 0.165
	MdUGT91AJ4	1.00 ± 0.104	2.451 ± 0.187	3.584 ± 0.224*	5.623 ± 0.295*	2.575 ± 0.193
	MdUGT91AJ5	1.00 ± 0.095	3.287 ± 0.194*	5.636 ± 0.317*	6.187 ± 0.286**	2.573 ± 0.167
	MdUGT91AJ6	1.00 ± 0.098	2.174 ± 0.156	2.652 ± 0.195	4.293 ± 0.313*	2.049 ± 0.209
	MdUGT91AJ7	1.00 ± 0.112	3.576 ± 0.178*	4.965 ± 0.282*	6.027 ± 0.306**	3.857 ± 0.216*
	MdUGT91C7	1.00 ± 0.106	2.041 ± 0.117	4.593 ± 0.315*	6.284 ± 0.392**	3.596 ± 0.223*
Mesotrione	MdUGT91AJ1	1.00 ± 0.113	2.194 ± 0.129	3.857 ± 0.237*	5.266 ± 0.287*	3.953 ± 0.285*
	MdUGT91AJ2	1.00 ± 0.107	1.746 ± 0.135	2.472 ± 0.225	4.593 ± 0.312*	2.491 ± 0.191
	MdUGT91AJ3	1.00 ± 0.114	1.375 ± 0.118	1.928 ± 0.238	2.572 ± 0.297	2.104 ± 0.188
	MdUGT91AJ4	1.00 ± 0.106	1.479 ± 0.127	2.586 ± 0.314	1.584 ± 0.326	1.206 ± 0.215
	MdUGT91AJ5	1.00 ± 0.102	2.958 ± 0.236	4.056 ± 0.298*	6.727 ± 0.305**	3.924 ± 0.227*
	MdUGT91AJ6	1.00 ± 0.113	2.962 ± 0.198	5.975 ± 0.254*	3.952 ± 0.297*	2.587 ± 0.263
	MdUGT91AJ7	1.00 ± 0.109	4.690 ± 0.213*	8.257 ± 0.263**	6.275 ± 0.313**	3.593 ± 0.285*
	MdUGT91C7	1.00 ± 0.106	3.254 ± 0.227*	5.285 ± 0.281*	4.205 ± 0.257*	3.286 ± 0.291*
Tembotrione	MdUGT91AJ1	1.00 ± 0.112	2.104 ± 0.190	3.575 ± 0.275*	4.198 ± 0.195*	3.271 ± 0.272*
	MdUGT91AJ2	1.00 ± 0.107	1.953 ± 0.115	2.756 ± 0.292	4.193 ± 0.286*	2.757 ± 0.186
	MdUGT91AJ3	1.00 ± 0.106	1.578 ± 0.138	2.143 ± 0.256	3.745 ± 0.225*	3.209 ± 0.245*
	MdUGT91AJ4	1.00 ± 0.109	1.987 ± 0.152	3.246 ± 0.293*	3.985 ± 0.215*	2.676 ± 0.220
	MdUGT91AJ5	1.00 ± 0.107	1.756 ± 0.147	2.445 ± 0.195	2.098 ± 0.192	1.545 ± 0.123
	MdUGT91AJ6	1.00 ± 0.104	2.465 ± 0.192	3.479 ± 0.224*	5.173 ± 0.244*	4.298 ± 0.274*
	MdUGT91AJ7	1.00 ± 0.106	2.284 ± 0.184	3.465 ± 0.272*	4.195 ± 0.191*	2.467 ± 0.207
	MdUGT91C7	1.00 ± 0.102	1.457 ± 0.165	1.920 ± 0.295	2.263 ± 0.183	1.479 ± 0.212

Bicyclopyrone	MdUGT91AJ1	1.00 ± 0.094	1.376 ± 0.129	1.457 ± 0.313	2.487 ± 0.176	2.244 ± 0.205
	MdUGT91AJ2	1.00 ± 0.110	1.598 ± 0.118	2.863 ± 0.267	3.556 ± 0.165*	2.299 ± 0.198
	MdUGT91AJ3	1.00 ± 0.106	2.487 ± 0.125	4.575 ± 0.301*	4.187 ± 0.181*	3.274 ± 0.186*
	MdUGT91AJ4	1.00 ± 0.128	2.101 ± 0.131	2.587 ± 0.297	3.562 ± 0.175*	2.495 ± 0.234
	MdUGT91AJ5	1.00 ± 0.115	1.495 ± 0.146	2.084 ± 0.286	2.849 ± 0.214	2.126 ± 0.226
	MdUGT91AJ6	1.00 ± 0.102	2.047 ± 1.756	2.576 ± 1.83	3.172 ± 1.96*	2.194 ± 0.213
	MdUGT91AJ7	1.00 ± 0.113	2.585 ± 0.256	2.948 ± 0.272	3.564 ± 0.282*	2.197 ± 0.198
	MdUGT91C7	1.00 ± 0.107	2.137 ± 0.224	3.576 ± 0.291*	4.058 ± 0.315*	2.572 ± 0.284
